# Meiotic drive is associated with sexual incompatibility in *Neurospora*

**DOI:** 10.1111/evo.14630

**Published:** 2022-10-03

**Authors:** Aaron A. Vogan, Jesper Svedberg, Magdalena Grudzinska‐Sterno, Hanna Johannesson

**Affiliations:** ^1^ Department of Organismal Biology Uppsala University Uppsala SE‐75236 Sweden; ^2^ Department of Biomolecular Engineering, Genomics Institute UC Santa Cruz Santa Cruz California 95064; ^3^ The Royal Swedish Academy of Sciences and Department of Ecology Environment and Plant Sciences, Stockholm University Stockholm SE‐106 91, California Sweden

**Keywords:** Bateson‐Dobzhansky‐Muller incompatibility, meiotic drive, *Neurospora*, sexual incompatibility, speciation

## Abstract

Evolution of Bateson‐Dobzhansky‐Muller (BDM) incompatibilities is thought to represent a key step in the formation of separate species. They are incompatible alleles that have evolved in separate populations and are exposed in hybrid offspring as hybrid sterility or lethality. In this study, we reveal a previously unconsidered mechanism promoting the formation of BDM incompatibilities, meiotic drive. Theoretical studies have evaluated the role that meiotic drive, the phenomenon whereby selfish elements bias their transmission to progeny at ratios above 50:50, plays in speciation, and have mostly concluded that drive could not result in speciation on its own. Using the model fungus *Neurospora*, we demonstrate that the large meiotic drive haplotypes, *Sk‐2* and *Sk‐3*, contain putative sexual incompatibilities. Our experiments revealed that although crosses between *Neurospora intermedia* and *Neurospora metzenbergii* produce viable progeny at appreciable rates, when strains of *N. intermedia* carry *Sk‐2* or *Sk‐3* the proportion of viable progeny drops substantially. Additionally, it appears that *Sk‐2* and *Sk‐3* have accumulated different incompatibility phenotypes, consistent with their independent evolutionary history. This research illustrates how meiotic drive can contribute to reproductive isolation between populations, and thereby speciation.

Throughout the course of the last century, a significant amount of progress has been made in understanding how evolution creates diversity in the natural world, and what factors drive speciation. It is understood that the phenomena controlling phenotypic divergence must coincide with changes at the genotypic level, and in terms of speciation, this divergence is often thought to be precipitated by so called Bateson‐Dobzhansky‐Muller (BDM) incompatibilities. In brief, the BDM model proposes that when two populations become isolated, changes at the genic level occur in such a way as for each population to evolve coadapted alleles. When these two populations meet, the gene products from the different populations are no longer able to perform their function and attempts to mate result in inviable or sterile hybrid offspring. Despite the strong theoretical framework around the BDM model, empirical data on gene interactions that cause reproductive isolation between populations remain relatively rare (Maheshwari and Barbash 2011). As a result, the exact processes that lead to the formation of BMD incompatibilities are also poorly understood.

Meiotic drive is the phenomenon where a selfish genetic element is able to manipulate the sexual cycle of an organism such that it becomes overrepresented in the offspring. Due to the mechanisms through which drivers cheat meiosis, there is often an associated penalty to fertility through the death of meiotic products, such as sperm or spores (Lindholm et al. 2016). For many of the described systems of meiotic drive, multiple genes are required for the drive to function, and these are kept in linkage through genomic inversions to suppress recombination (Presgraves 2009), which effectively isolates the driving haplotype from the nondriving version and can lead to the accumulation of linked deleterious mutations (Dyer et al. 2007). These regions of suppressed recombination are often large and encompass many genes, both related and unrelated to drive, which sets the stage for BDM incompatibilities to form. In agreement with this expectation, many cases have been described where individuals carrying meiotic drive elements show reproductive incompatibilities with nondriving individuals (Wilkinson and Fry 2001; Phadnis and Orr 2009; Zhang et al. 2015). Such incompatibilities may ultimately result in the inability of a driver to sweep to fixation in a population, as for driving alleles to reap the benefits of cheating meiosis, they must reproduce with naïve individuals. Alternatively, the reproductive isolation caused by the drive could effectively split the population and result in speciation. This latter outcome has been under considerable debate, particularly for sex‐biased drives, with opinions regarding the ability and importance of drivers to cause speciation varying wildly (Frank 1991; Coyne and Orr 1993; Zhang et al. 2015; Sweigart et al. 2019). Thus, additional empirical data are required to address the role of meiotic drive in speciation.

The filamentous fungus *Neurospora crassa* and other closely related *Neurospora* species have been developed as a model system for comparisons of species recognition criteria and for the study of the evolution of reproductive isolation between species (Dettman et al. 2003a, 2003b, 2006; Menkis et al. 2009; Villalta et al. 2009; Turner et al. 2011). They also harbor meiotic drivers. The three meiotic drivers known in *Neurospora* are *Sk‐1*, *Sk‐2*, and *Sk‐3* and these function by means of spore killing, whereby spores that carry the driving element (i.e., the killer gene) will kill their sibling spores that do not (Vogan et al. 2022). Spore killing has a very obvious phenotype in *Neurospora*. Following meiosis, one round of mitosis occurs to produce eight sexual spores (ascospores). These spores are packaged together in an ascus to form a single row. When spore killing occurs, four viable, black ascospores are observed together with four small aborted white ascospores in each ascus (Turner and Perkins 1979). *Sk‐2* and *Sk‐3* are multi‐gene drivers that were both discovered in the species *Neurospora intermedia*. In this species, the killing gene is linked to the resistance gene in a haplotype that extends across a 30‐cM region surrounding the centromere on chromosome 3, resulting in a region of linkage covering roughly 400 genes or 2.5 Mbp (Svedberg et al. 2018). The two spore killers are mutual killers, meaning they produce no viable spores when crossed to each other, which is in contrast to crosses between two *Sk‐2* strains (or two *Sk‐3* strains), which result in eight viable spores, that is, no spore killing. *Sk‐2* and *Sk‐3* use different alleles of the same resistance gene and occupy a similar region of chromosome 3 (Hammond et al. 2012), but do not share the same killing gene and appear to have had a long history of evolutionary separation (Svedberg et al. 2018; Rhoades et al. 2019).

Turner (2001) surveyed available *Neurospora* strains for the presence of spore killing, and during this work discovered that some sensitive strains of *N. intermedia* were sterile in crosses with *Sk‐2* and *Sk‐3* spore killer strains. A subset of these strains was later reclassified as *N. metzenbergii* (Dettman et al. 2003a; Villalta et al. 2009), suggesting the possibility that BDM incompatibilities may have accumulated in the *Sk‐2* and/or *Sk‐3* spore killing haplotype of *N. intermedia*. To investigate this hypothesis, we first used molecular markers (Dettman et al. 2003a) to determine the phylogenetic placement of the *N. intermedia* strains that showed sterility in crosses to *Sk‐2* and *Sk‐3* and determined that these strains do in fact belong to the sister species *N. metzenbergii* (Dettman et al. 2003a). Subsequently, by analyzing reproductive success in laboratory crosses, we verified that *N. metzenbergii* strains display a higher degree of reproductive isolation with *N. intermedia* strains carrying *Sk‐2* or *Sk‐3* than with those that do not. Additionally, we found that the mechanism of reproductive isolation appears to differ between *Sk‐2* and *Sk‐3*, suggesting that the separate drivers have captured unique incompatibility factors during their independent evolutionary separation. We conclude that the meiotic drive haplotypes carry genes conferring strong reproductive isolation between strains of *Neurospora*, either by pleiotropic effects of the killer genes themselves or by the action of other linked genes, and discuss the role that meiotic drive can play in speciation.

## Methods

### STRAINS

*Neurospora* strains used in this study were acquired from the Fungal Genetics Stock Center (FGSC; fgsc.net). All strains annotated as *N. metzenbergii* (*n* = 17) in the FGSC collection were included in the analysis, as well as all strains annotated as *N. intermedia* that were collected from New Zealand (*n* = 11) or Mexico (*n* = 1). Additional strains of *Neurospora* (*n* = 54) were chosen to represent the diversity of the species based on Dettman et al. (2003a). We also included in our study strains of *N. intermedia* carrying either *Sk‐2* or *Sk‐3*. Note that for strain 7427 and 7428 (which contain the *Sk‐2* allele) and strains 3193 and 3194 (containing the *Sk‐3* allele), the original strains that carried the spore killer haplotype are not available from the FGSC. Instead, these *Sk‐2* strains are F1 progeny of the wild spore killer strain and either of the *N. intermedia* tester strains 1766 or 1767, and the *Sk‐3* strains are the result of three backcrosses from the original wild spore killer strains to the 1767 strain. Strain 7426 represents a wild strain that carries *Sk‐2*, which was isolated at a later date. All strains used for mating assays are referred to by their FGSC ID number and are listed in Table S1. Strains 8761 and 8762 are single conidial isolates derived from 1766 and 1767, respectively. Locations of strains from the Perkin's collection were taken from the FGSC (www.FGSC.net) and converted to global coordinates using the R package ggmap (Kahle and Wickham 2013).

### MOLECULAR MARKERS AND PHYLOGENETIC ANALYSES

As molecular markers, we used four unlinked anonymous nuclear loci (TMI, TML, DMG, and QMA) that were identified in Dettman et al. (2003a) as suitable for species delimitation in *Neurospora*. We PCR‐amplified and Sanger sequenced the markers according to the protocols given in Dettman et al. (2003a), to assign the investigated strains to species‐level groupings. These markers contain microsatellite sequences, which were removed prior to alignment, and hence only the flanking, nonrepetitive, sequences were aligned and analyzed, as in Dettman et al. (2003a). When comparing our sequencing results of a number of reference strains to those sequences generated by Dettman et al. (2003a) and subsequently used by Villalta et al. (2009), we discovered a large number of cytosines in the Dettman et al. (2003a) sequences that are absent from ours, as well as from sequences from Corcoran et al. (2014) and Svedberg et al. (2018). Due to these discrepancies, we only used marker sequences that we generated ourselves in this study, or that were generated by Corcoran et al. (2014) (Table S2). Sequences with the microsatellite portions removed were concatenated and aligned with the CLC sequence viewer version 7.7 (https://digitalinsights.qiagen.com/), followed by manual curation. The final alignment of 71 sequences had a length of 2093 columns, 915 distinct patterns, 372 parsimony‐informative, 228 singleton sites, and 1493 constant sites. We performed a Maximum Likelihood analysis using IQ‐TREE version 2.0 (Minh et al. 2020) with the default parameters and 100 standard nonparametric bootstraps. The TPM3+F+R2 model was selected based on the Bayesian information criterion. Sequences are deposited in GenBank with accession numbers MW034381–MW034436.

Additionally, we performed a phylogenomic analysis based on SNP data from 40 strains distributed over eight species of *Neurospora* (Table S2). Genomic data for these strains had been collected in previous studies (Corcoran et al. 2014; Svedberg et al. 2018) and SNPs were called against the *N. intermedia* strain 8807. For details on SNP calling, see Svedberg et al. (2018). Whole chromosome phylogenies based on a total of 4,680,447 variable sites were inferred using RAxML (Stamatakis 2014) for six of the seven chromosomes separately, using the following parameters: raxmlHPC‐HYBRID‐AVX ‐T 16 ‐m GTRCAT ‐x 45345 ‐p 22455 ‐# 100 ‐f a. Chromosome 3 was excluded to remove the conflicting phylogenetic signal of the nonrecombining spore killer region compared to the rest of the genome, even though that only affects the placement of strains within *N. intermedia*. The six phylogenetic trees (bipartition trees generated by RAxML) were then merged into a consensus network with 20% pruning, using Splitstree (Huson and Bryant 2006).

### CROSSINGS

All crosses were conducted in 10 × 100 mm glass culture tubes containing 1 mL of liquid synthetic crossing media (Westergaard and Mitchell 1947) with no added sucrose. A small strip of filter paper was added to the tubes before autoclaving. *Neurospora intermedia* strains were used as females for all hybrid crosses: they were inoculated into the tubes and allowed to establish and produce protoperithecia (immature fruiting bodies) over 3 days at room temperature in complete darkness. Conidia (asexual spores that also function as fertilizing agents) from strains used as males in the crosses were then used to fertilize the protoperithecia and after fertilization, the cultures were incubated at 25°C in a 12 h light/dark cycle. After 2–3 weeks, mating had taken place for sexually compatible crosses, perithecia (mature fruiting bodies) were formed, and ascospores had been shot, as evidenced by empty perithecia. To generate offspring from crosses, ascospores were harvested from the walls of the culture tubes using a sterile loop and spread onto the surface of water agar plates. These plates were incubated in a water bath at 60°C for 1 h to induce germination as *Neurospora* ascospores require a heat treatment to germinate. This treatment kills any contaminating asexual spores (conidia) or mycelia, but will also kill nonpigmented ascospores. Germinated spores were picked from the water agar and placed into sterile glass culture tubes with Vogel's media (Vogel 1956) to establish cultures.

### ESTIMATION OF REPRODUCTIVE SUCCESS

To measure the reproductive success of crosses between strains of *Neurospora*, we evaluated the production of perithecia and estimated the proportion of shot black spores to unpigmented white/hyaline spores, following Dettman et al. (2003b). In brief, crosses were allowed to mate until all ascospores had been shot. The sides of the culture tubes were examined under a dissecting microscope at 250× magnification and the proportion of black ascospores was estimated. The results of crosses were scored on a scale from 0 to 6 following Dettman et al. (2003b), where 0 refers to crosses that were entirely sterile (no fruiting bodies produced) and 6 refers to a fully compatible cross (fruiting bodies were produced and shot many black ascospores). The presence of an ostiole was not recorded as a measure of reproductive compatibility; hence, categories 1 and 2 used by Dettman et al. (2003b) are merged in this study. For selected combinations, we set up the crossings in triplicate to determine final values. To verify the accuracy of the method and obtain quantitative values, proportions of black spores were also estimated through perithecial dissections for a limited number of crosses. For this method, we investigated the crosses at an earlier developmental stage, prior to the ejection of ascospores. Mature perithecia were identified based on the presence of long extended ostioles and selected for dissection. Perithecia were dissected on glass slides in a drop of sterile water to obtain rosettes of asci. Only rosettes that contained at least some fully matured black spores were evaluated for the proportion of viable spores. Additionally, perithecia were harvested over a 1‐week period to verify that spore production was evaluated at the optimal time, that is, after most asci had matured, but before most of the ascospores had been shot.

## Results

### PHYLOGENETIC PLACEMENT OF REPRODUCTIVELY ISOLATED STRAINS

**Figure 1:**
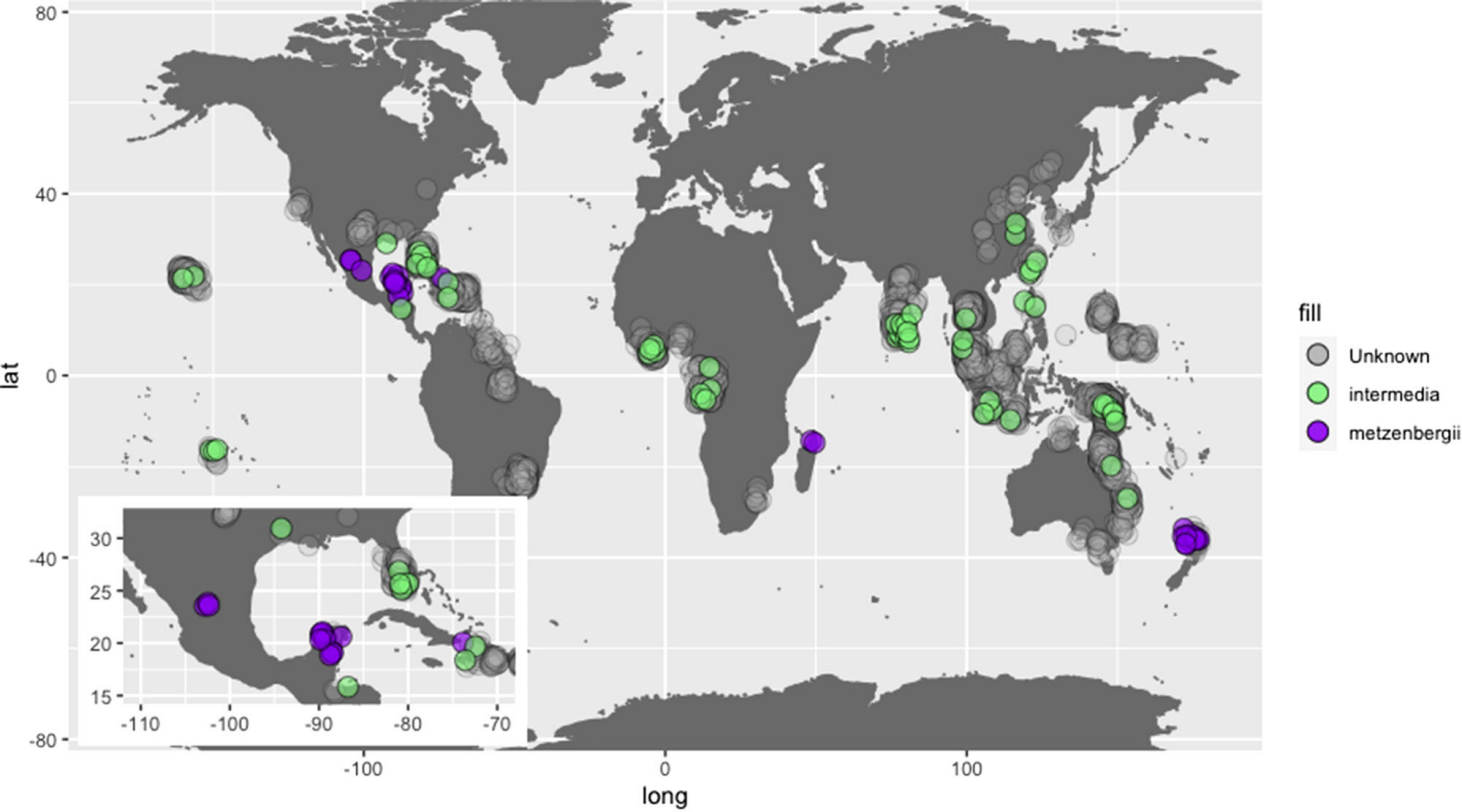
Global distribution of *Neurospora intermedia* and *Neurospora metzenbergii*. All strains from the Perkins collection at FGSC (http://www.fgsc.net) that were determined to be *N. intermedia* through crossing to reference strains are plotted. The geographic origins of those which were confirmed as *N. intermedia* through molecular evidence are shown in green, and those which were revealed to be *N. metzenbergii* are shown in purple. The “unknown” strains refer to isolates of *N. intermedia* with no molecular data. The inset is a magnified view of Mexico and surrounding regions. Note that strains with no precise locale data are visualized as midpoints in their country of origin, including three in Mexico.

In total, 3038 wild strains of *Neurospora* have been collected and annotated as *N. intermedia* based on compatible matings when crossed with reference strains by Dr. David Perkins and others. The details of these crosses are publicly available from the FGSC (http://www.fgsc.net/Neurospora/PerkinsWildCollectionatFGSC.xlsx), and many of the strains are maintained there as well. Strains from New Zealand and Mexico were previously highlighted as mating particularly poorly to spore killers strains (Turner 2001), thus we obtained all available strains from these two locales that are annotated as *N. intermedia* or *N. metzenbergii* and determined their phylogenetic placement as per Dettman et al. (2003a). This analysis determined that all strains originally assigned to *N. intermedia* and showing poor reproduction with spore killers belong to the species *N. metzenbergii* (Fig. S1), a result that was confirmed with a phylogenomic approach (Fig. S2). This analysis also reveals an interesting biogeographic pattern where it appears that although *N. intermedia* has a global distribution, it never co‐occurs with *N. metzenbergii* (Fig. 1). This nonoverlapping distribution is most striking around Mexico, where all isolates were determined as *N. metzenbergii*, whereas *N. intermedia* were found immediately to the north, in Texas, and to the south, in Honduras. The one exception to the nonoverlapping distribution is strain 10399 of *N. metzenbergii* that was isolated from Haiti alongside *N. intermedia* strains (Fig. 1).

### THE SPORE KILLERS OF *N. intermedia* SHOW HIGHER INCOMPATIBILITY WITH *N. metzenbergii*

Previous work established that *N. metzenbergii* and *N. intermedia* are partially reproductively isolated, in that they produce a lower number of viable offspring when crossed to each other as opposed to crosses among strains of their own species (Dettman et al. 2003b; Villalta et al. 2009). We verified that the newly assigned NZ strains of *N. metzenbergii* show similar levels of reproductive isolation to *N. intermedia* by crossing representative strains of *N. metzenbergii* to standard tester strains of *N. intermedia* and among themselves. We see that, as expected, for both species the within‐species crosses resulted in a high proportion of viable ascospores (>90%; Table S3). Furthermore, most strains of *N. metzenbergii* were able to mate with the *N. intermedia* tester strains (1766 and 1767), but produced consistently low proportions of viable spores (60%–80%). Notable exceptions to this range were the *N. metzenbergii* strain 10399 from Haiti, which produced even fewer viable spores (30%–60%), and 7829 from New Zealand, which did not mate with any *N. intermedia* strains (Table S3). Previous studies have also shown that some populations of *N. intermedia* have evolved strong premating barriers with *N. crassa* (Turner et al. 2011). We investigated two strains from these populations here, 2316 and 6263, which revealed that these barriers may also prevent mating of these *N. intermedia* strains with strains of *N. metzenbergii* as successful matings were only achieved with one *N. metzenbergii* strain, 5120 (Table S3 and S4).

In contrast to crosses with tester strains, crosses of the *N. metzenbergii* strains to the strains of *N. intermedia* that carry either *Sk‐2* or *Sk‐3* showed a dramatic reduction in the proportion of shot black spores. A drop of 50% in the proportion of viable spores is expected due to the spore killing itself; however, many of the crosses resulted in nearly no black spores, or failure to produce perithecia at all, suggesting that there are strong incompatibilities within the spore killer region (Table S3). To confirm that spore killing was occurring in these interspecific crosses, perithecia were dissected to screen for the standard 4:4 white to black spore pattern that should be observed. For all crosses between the spore killer strains and *N. metzenbergii* strains, the asci mostly contained either small aborted white spores, misshapen black spores, or white spores. In general, few asci were observed that could be properly evaluated for spore killing, but some did appear to have the standard 4:4 pattern, indicating that spore killing is active in the interspecific crosses.

**Figure 2:**
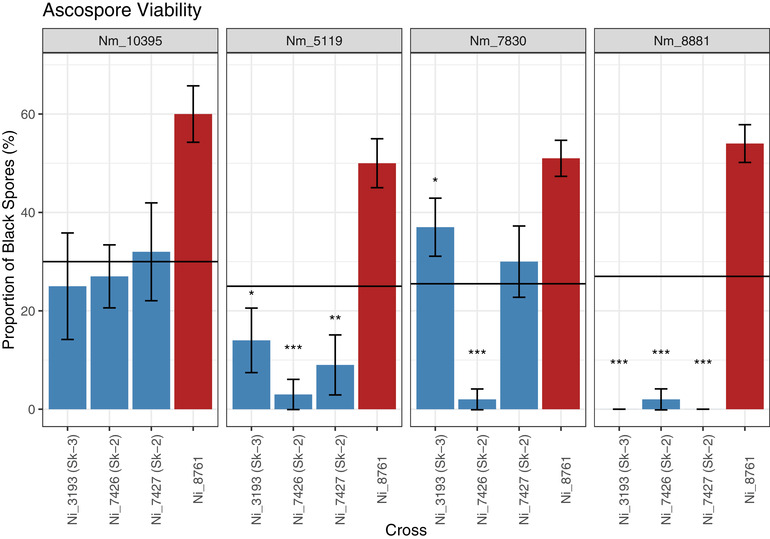
Proportion of black spores produced by crosses between four *Neurospora metzenbergii* strains (10395 [Mexico], 5119 [New Zealand], 7830 [New Zealand], and 8881 [Madagascar]) and four *N. intermedia* strains (3193 [*Sk‐3*], 7426 [*Sk‐2*], 7427 [*Sk‐2*], and 8761 [sensitive]). Horizontal lines represent half the value of the cross to 8761 to the given *N. metzenbergii* strain as an expectation for a decrease in germination due to spore killing alone. Asterisks represent significant deviations from this expectation according to a chi square test (* <0.05, ** <0.01, *** <0.001); whiskers denote one standard error.

Additionally, we used perithecial dissections of a subset of strains to quantify the degree of reproductive incompatibility (Table S4). For all interspecific crosses, few asci were observed to have a full component of eight black ascospores. Nearly all asci contained at least one small aborted ascospore, and some asci appeared to be entirely aborted. Large proportions of partially pigmented ascospores or brown spores were also observed. Categorizing brown spores within asci is difficult, as these spores may have still developed into black spores prior to shooting. Additionally, some number of brown spores are viable and survive the heat treatment to germinate (Ho 1986). Due to these caveats, brown spores were considered viable in the data presented here. The perithecial dissections confirmed that some of the crosses of *N. metzenbergii* strains to *Sk‐2* and *Sk‐3* strains show a dramatic reduction in black spore production as compared to crosses with non‐spore killer strains. Strains 10395 and 7830 did not exhibit a decrease in the proportion of black spores that was greater than the 50% expected from spore killing alone when crossed to the lab strains of *Sk‐2* and *Sk‐3*; however, the methods used here may ignore completely aborted asci and so only quantify within‐ascus incompatibility. Strains 5119 and 8881 both show a considerable reduction in the proportion of black spores produced, with 8881 being the most severe. Crosses to the wild *Sk‐2* strain 7426 were similar to the lab‐derived strains for 10395, but strains from NZ showed a large decrease in black spore production, suggesting that there may be additional incompatibilities in the non‐spore killing region that have been lost in the lab‐derived strains (Fig. 2).

### DIFFERENT INCOMPATIBILITY PHENOTYPES OF *Sk‐2* AND *Sk‐3* WITH *N. metzenbergii*

**Figure 3:**
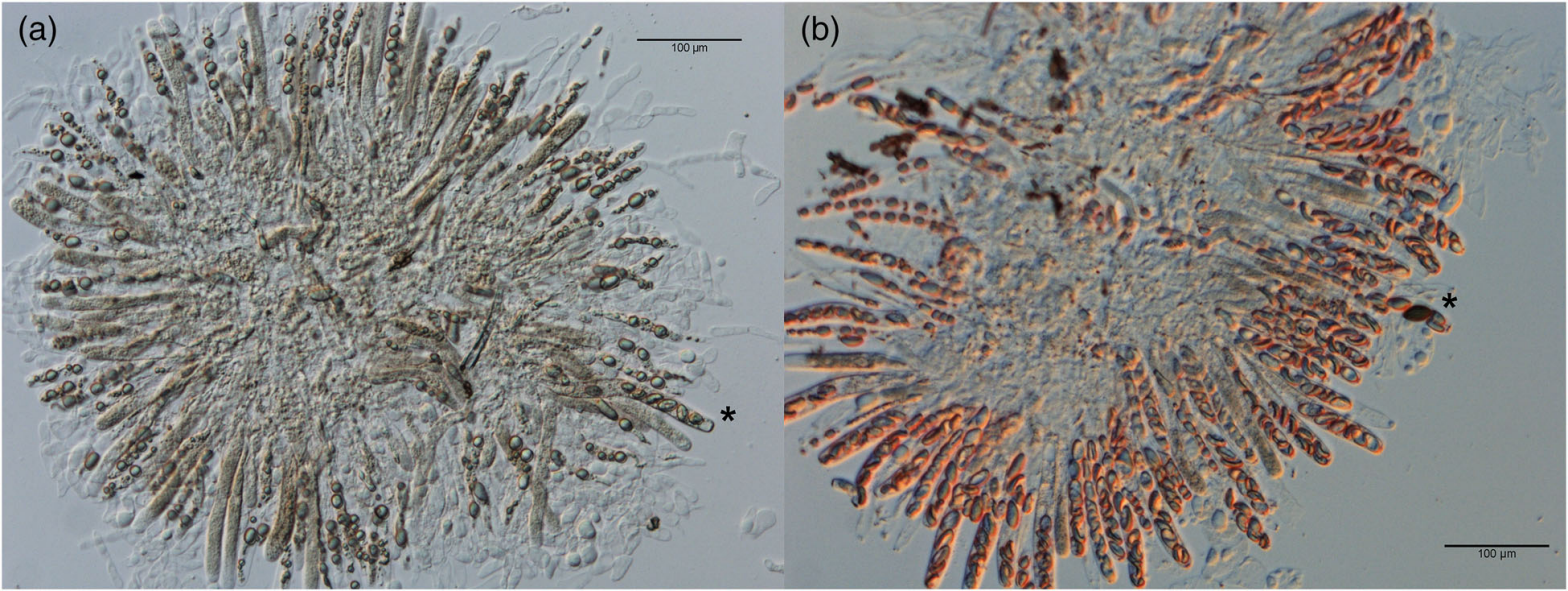
Rosettes of asci from crosses between *Neurospora metzenbergii* 8881 and (a) the *Sk‐2* backcross strains or (b) the *Sk‐3* backcross strain. For crosses to *Sk‐2*, nearly all asci are aborted without producing spores of any kind, but occasionally will produce an ascus with small aborted spores (*). For *Sk‐3* crosses, asci contain only small aborted spores most of the time, but occasionally viable spores are found (*).

A number of *N. metzenbergii* strains displayed different incompatibility phenotypes when crossed to *Sk‐2* than when crossed to *Sk‐3* despite the fact that the genomic background of the spore killer strains should be highly similar outside of the nonrecombining spore killer region. With the Madagascar strains, 8880 and 8881, and strain 8846 (Mexico), no spores are shot when crossed to the *Sk‐2* strains, but many small aborted white spores are shot when crossed to the *Sk‐3* strains, suggesting that there are different incompatibilities present within the different spore killer regions. To confirm that the spore killing region is specifically associated with the reproductive isolation observed between the Madagascar strains and the *Sk‐2* and *Sk‐3* strains, F1 progeny from crosses between 1767 and either 3193 (*Sk‐3*) or 7427 (*Sk‐2*) was backcrossed four times to 1766 to generate spore killer strains with a more isogenic background to 1766. Crosses to these 5× backcrossed strains showed identical phenotypes as to the parental spore killer strains, confirming the association of the phenomenon to the spore killer region. To verify these phenotypes, perithecial dissections were conducted. These confirmed that perithecia appear to be formed normally (ostioles are present) in crosses to both spore killers, but that in the *Sk‐2* crosses, most asci abort at the spore‐forming stage (Fig. 3a), whereas in the *Sk‐3* crosses, spores are formed, but nearly all are abortive (small and white) (Fig. 3b). With crosses to *Sk‐3*, occasionally viable spores can be found (Fig. 3b), and these can germinate after heat treatment, showing they are fully viable. Given this low rate of viable spore production, it is likely due to rare recombination events within the spore killer region, which is hypothesized to occur occasionally (Svedberg et al. 2018).

To investigate the incompatibilities further, hybrids were collected from crosses between the Madagascar strain 8880 and the *N. intermedia* strains 8761 and 8795, and crossed to the *Sk‐2* and *Sk‐3* strains. Most of the hybrids maintain the specific incompatibilities when crossed to *Sk‐2* and *Sk‐3* tester strains; however eight out of 36 recover some fertility. A few strains show differential recovery of fertility between *Sk‐2* and *Sk‐3*, in that some black spores were observed in crosses to the *Sk‐2* strain, but not the *Sk‐3* strain, or vice versa, supporting the hypothesis that the different phenotypes observed between *Sk‐2* and *Sk‐3* crosses represent different underlying incompatibilities.

## Discussion

Here, we provide evidence that meiotic drive in *Neurospora* contributes to reproductive isolation, both directly through killing half of all spores and through the accumulation of additional incompatibilities, a process that is often overlooked in discussions of drive systems and speciation (Sweigart et al. 2019). Although the low frequency of both drive and full incompatibility suggests that the precise incompatibilities at play in *Sk‐2* and *Sk‐3* are unlikely to have been major contributors in the divergence of *N. metzenbergii* and *N. intermedia*, it nevertheless provides an example of a scenario where meiotic drive could lead to speciation in other systems.

To understand what conditions may have led to the evolution of reproductive incompatibilities between *N. intermedia* and *N. metzenbergii*, it is important to determine what evolutionary forces were likely most dominant during their divergence. The delimitation between *N. intermedia* and *N. metzenbergii* aligns well with the recognition criteria for both the biological species concept and the phylogenetic species concept, in that molecular markers demonstrate their monophyly and mating assays show them to reproduce better with conspecifics than interspecifics (Dettman et al. 2003a, 2003b). Here, we have demonstrated that strains of *Neurospora* that were isolated from New Zealand, and previously designated as *N. intermedia*, in fact belong to *N. metzenbergii*, nearly doubling the number of known *N. metzenbergii* samples in collections and highlighting the unusual geographical distribution of the two species. This distribution is consistent with them competing to the end of competitive exclusion, but as strains were collected at different times, temporal factors cannot be eliminated. Together, these facts all point to the possibility that the *N. intermedia* and *N. metzenbergii* originally diverged allopatrically (species with overlapping ranges tend to compete less [Letcher et al. 1994]), thereby fulfilling the main requirement for the formation of BDM incompatibilities.

We have provided evidence that additional reproductive barriers exist within the spore killer region of *Sk‐2* and *Sk‐3* strains of *N. intermedia* that inhibit the ability of said strains to hybridize with *N. metzenbergii*. The reduced fertility is partially driven by the 50% loss of spores due to spore killing, but the strains carrying *Sk‐2* and *Sk‐3* also exhibit a strong reduction in fertility with some strains of *N. metzenbergii* that go beyond what can be explained by the activity of the spore killer elements alone. It could be expected that the reason crosses between the spore killers and *N. metzenbergii* strains show a decrease in viable progeny is the production of unbalanced gametes as a result of the mismatched inversions, but as a reduced fertility is not observed between crosses of spore killer and sensitive strains within *N. intermedia*, it is unlikely to be a significant issue. Additionally, the fact that the strongest incompatibilities with *N. metzenbergii* strains appear to be polymorphic further argues against a significant role of the inversions. The occurrence of polymorphic incompatibility factors between populations has been discussed as variable reproductive isolation (VRI), and has been observed in a variety of systems (Cutter 2012). Under a VRI scenario, these polymorphic alleles could originate either from ancestral, incomplete lineage sorting or as derived alleles within *N. metzenbergii*, but derived alleles are more likely to contribute to reproductive isolation (Orr 1995). Moreover, as only the killer haplotype is incompatible with these *N. metzenbergii* strains, the incompatible locus of *N. intermedia* must be located within the *Sk* region. Additionally, the differential phenotypes exhibited between *Sk‐2* and *Sk‐3* crosses suggest that discrete incompatible loci of large effect exist within the *Sk‐2* and *Sk‐3* regions. Conversely, as *Sk‐2* and *Sk‐3* do not appear to share the same killer gene (Rhoades et al. 2019) it could be possible that the spore killing genes themselves are responsible for the incompatibility through pleiotropic effects on reproduction, as has been observed with one meiotic driver from *Drosophila* (Phadnis and Orr 2009). Whether the incompatibility is caused by the killer gene or another gene confined within the haplotype needs further investigation.

The fact that incompatibilities are observed to accumulate in the nonrecombining killer haplotype potentially provides deeper insights into the evolution of BDM incompatibilities. Although the per base pair rate of divergent substitutions between *N. intermedia* and *N. metzenbergii* is higher in the *Sk* region than the rest of the genome, these still represent the minority of total divergent sites across the genome (10,345/191,911 substitutions) and the spore killer strains, in fact, have slightly fewer nonsynonymous substitutions in total than sensitive strains (59,077 vs. 63,279) (Svedberg et al. 2018). It is known that selection plays an important role in the accumulation of BDM incompatibilities and although a lot of attention has been paid to directional selection and stabilizing selection (Welch 2004; Fierst and Hansen 2010; Nosil and Schluter 2011), less has been given to decreased purifying selection. Additionally, a considerable amount of theory and modeling have discussed the role of deleterious recessive mutations linked to drivers or regions of reduced recombination in speciation (Navarro and Barton 2003; Welch 2004; Kirkpatrick and Barton 2006). We can be confident that deleterious recessive mutations are not a factor in *Sk‐2* or *Sk‐3* as crosses among killer strains of the same type show no decrease in the production of viable offspring. Nonetheless, the spore killer regions may be accumulating nonfunctional mutations due to the relaxed selection in the *Sk* haplotype. Importantly, only nonfunctional mutations that can be compensated for by the genomic background of *N. intermedia* will persist, as others will be highly deleterious or lethal. However, in the hybrid crosses to *N. metzenbergii*, alleles allowing for such compensation may not be present due to recombination and/or independent assortment of chromosomes. So, although phylogenomic analyses suggest that most sites in the *Sk* haplotype are diverging under neutral expectation, the region may be a hotspot for the formation of BDM incompatibilities (Svedberg et al. 2018). These ideas should hold true for any genomic region experiencing suppressed recombination, such as inversions, and, for this reason, have broader implications beyond meiotic drive.

Under the alternative scenario where the killer genes themselves are responsible for the sexual incompatibilities, a different model may explain the observations. Instead of BDMs being more likely to form in the nonrecombining haplotype, it is possible that spore killer genes are involved in pathways related to sexual reproduction, and that genes in these pathways are more prone to become BDM incompatibilities. This appears to be the case with *Overdrive* in *Drosophila pseudoobscura*, where *Overdrive* is a meiotic drive gene, but also has many downstream trans targets that it interacts with. It has accumulated more nonsynonymous mutations than any of its targets, suggesting it may be diverging faster than the background rate (Phadnis and Orr 2009). The molecular mechanisms through which *Sk‐2* and *Sk‐3* operate are largely unknown, although in the case of *Sk‐2* it does not appear to be directly acting through a disrupted sexual cycle, as transformants can be constructed that kill during vegetative growth (Rhoades and Hammond 2021). Whatever the cause of the observed sterility in crosses to *N. metzenbergii*, it must be specific to the *N. metzenbergii* background, as both *Sk‐2* and *Sk‐3* do not show similar patterns when crossed to the more distantly related *N. crassa* (Turner and Perkins 1979).

Hybrid incompatibilities have been linked to meiotic drivers in numerous systems (Braidotti and Barlow 1997; Tao et al. 2001; Wilkinson et al. 2003; McDermott and Noor 2010), but the discussion of how they could contribute to speciation has largely centered around the co‐evolution of complex suppressor systems, and the divergence of those systems in disparate populations (Courret et al. 2019). This discussion may be largely irrelevant here as no suppressors are known to be involved in *Sk‐2* or *Sk‐3*, nor is there any evidence of other meiotic drivers within *N. metzenbergii*. With spore killing (and mechanistically similar meiotic drivers), if mutual killers, such as *Sk‐2* and *Sk‐3*, fix in different populations, then crosses between individuals of those populations will be entirely incompatible. Such a scenario appears to have arisen in the fission yeast, *Schizosaccharomyces pombe*, where the diverse group of *wtf* genes show unique distributions in individual lineages (Bravo Núñez et al. 2020). Mutual killing may be a common phenomenon as it is observed in three well‐described systems: *S. pombe*, *N. intermedia*, and for the *Spok* genes of *Podospora anserina* (Vogan et al. 2019). However, such a mechanism will only remain in place as long as the drive is active, if the driver reaches fixation in a given population, it may be prone to decay (as observed in both *S. pombe* and *P. anserina*) and the reproductive barrier that it caused would disappear. Therefore, cases where the driving mechanism and/or co‐evolved suppressor genes implicitly cause reproductive isolation may not be expected to persist through time to impact speciation. However, if BDM incompatibilities have time to form during this phase or, as discussed above, are even more prone to formation in these regions, then drive could still contribute significantly to the reproductive isolation between populations, even if said drive then subsequently vanishes from the populations.

It should be noted that both the *Spok* and *wtf* genes are single gene drivers, which should be much less prone to the accumulation of linked incompatibilities illustrated here. Additionally, these small loci could be prone to cross species boundaries, as is similarly observed in *Drosophila* (Meiklejohn et al. 2018). In fact, the third known spore killer in *Neurospora*, *Sk‐1* from *N. sitophila*, is also a single gene driver and has also been observed to jump species boundaries (Svedberg et al. 2021). In a scenario where reproductive isolation is caused by mutual killers, if an individual were able to acquire both drivers, it would be resistant to both drives and therefore viable with both species/populations. The selective advantage for such an individual could be quite strong, arguing against single gene drives as speciation genes. This implies that meiotic drivers that constitute large haplotypes may have greater impacts on population structure than single gene drives, and hence be observed more often during interspecies/population crossings. Therefore, although until recently, meiotic drive systems were thought to require multiple linked loci, it may be the case that single gene drivers are much more common but are observed less due to minimal linked phenotypes.

## AUTHOR CONTRIBUTIONS

AV designed and conducted experiments, ran analyses, made figures, and wrote the first draft. JS performed project inception, ran analyses, and edited drafts. MGS conducted experiments and edited drafts. HJ performed project inception, edited drafts, and provided funding.

## CONFLICT OF INTEREST

The authors declare no conflict of interest.

## DATA ARCHIVING

Sequences generated in this study are deposited in GenBank with accession numbers MW034381–MW034436.

Associate Editor: T. Bataillon

Handling Editor: P. T. Chapman

## Supporting information


**Supplementary Figure 1**. Maximum likelihood tree of 69 *Neurospora* strains, based on four genetic markers, TMI, TML, DMG, and QMA. *N. discreta* was set as the outgroup and the branch delineating the outgroup was shortened for illustration purposes. The spore killer strains are marked in red and blue for *Sk‐2* and *Sk‐3*, respectively. Sequences that were generated in this study are marked with an asterisk (*), all of which were formerly annotated as *N. intermedia*. The Nx prefixes prior to the FGSC numbers denote the species of the given strain.

**Supplementary Figure 2**. Network of the terminal *Neurospora* clade based on maximum likelihood trees inferred from whole chromosome SNP data from six of the seven chromosomes; Chromosome 3 carries the *Sk* locus and so was excluded from the analysis. Colored ovals denote species of *Neurospora*. Note that strain 4723 falls outside all currently delimited species and may represent an undescribed species. Strains carrying *Sk‐2* are marked with red text and, the one *Sk‐3* strain is marked in blue. Network pruning level was set to 20%.

**Supplementary Table 1**. Strains used in this study

**Supplementary Table 2**. Strains and data used for phylogenetic analyses

**Supplementary Table 3**. Proportion of shot black ascospores from laboratory crosses.

**Supplementary Table 4**. Proportion of black ascospores of laboratory crosses from dissected perithecia.
