# Supplementary figures and images for: Meiotic drive is associated with sexual incompatibility in Neurospora
Source: Evolution. 2022 Oct 3;76(11):2687–96. doi: 10.1111/evo.14630 (PMC9828778; doi:10.1111/evo.14630)

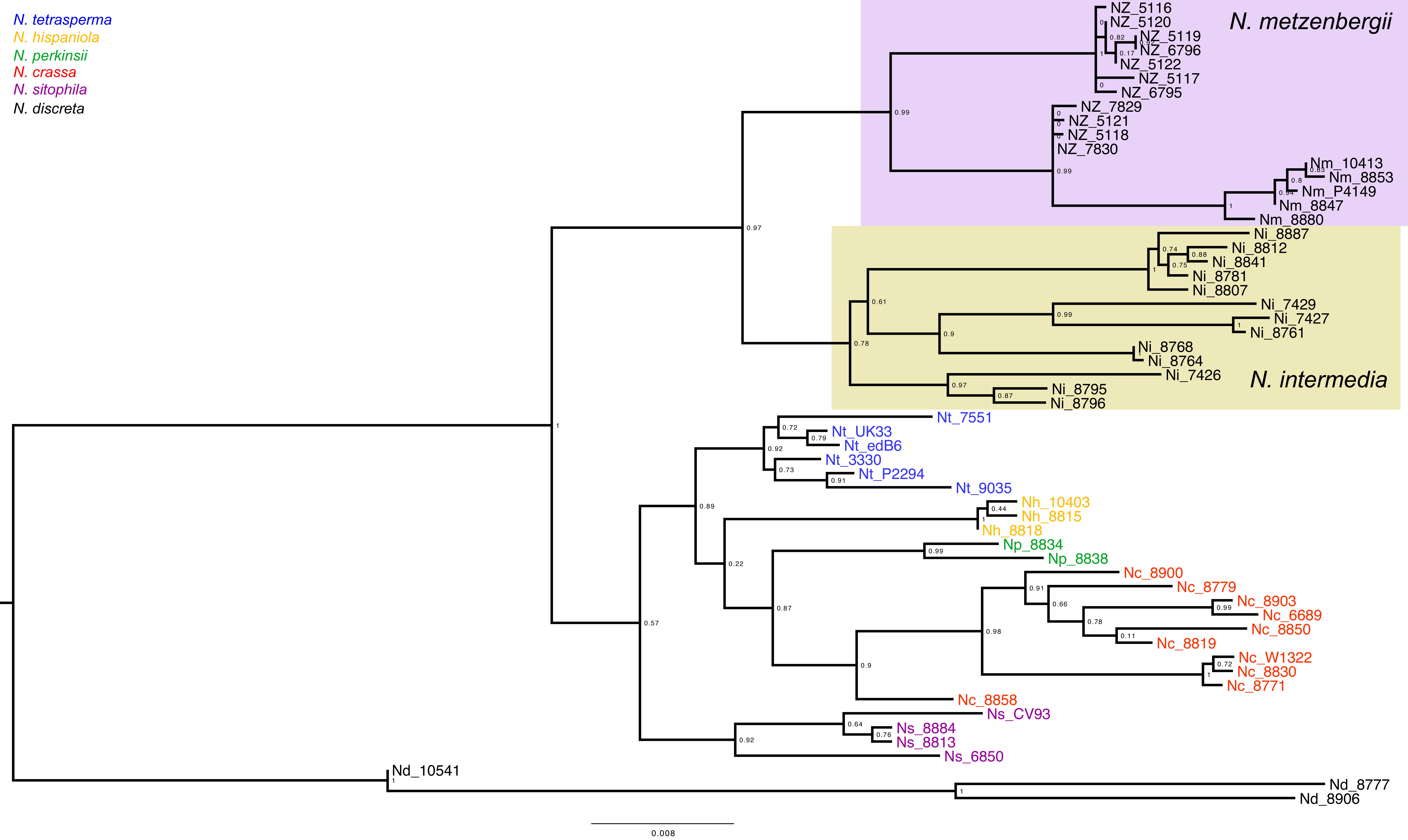

Supplement: Supplementary file 1 — Supplementary Figure 1. Maximum likelihood tree of 69 Neurospora strains, based on four genetic markers, TMI, TML, DMG, and QMA. N. discreta was set as the outgroup and the branch delineating the outgroup was shortened for illustration purposes. The spore killer strains are marked in red and blue for Sk‐2 and Sk‐3, respectively. Sequences that were generated in this study are marked with an asterisk (*), all of which were formerly annotated as N. intermedia. The Nx prefixes prior to the FGSC numbers denote the species of the given strain. [file EVO-76-2687-s003.png]

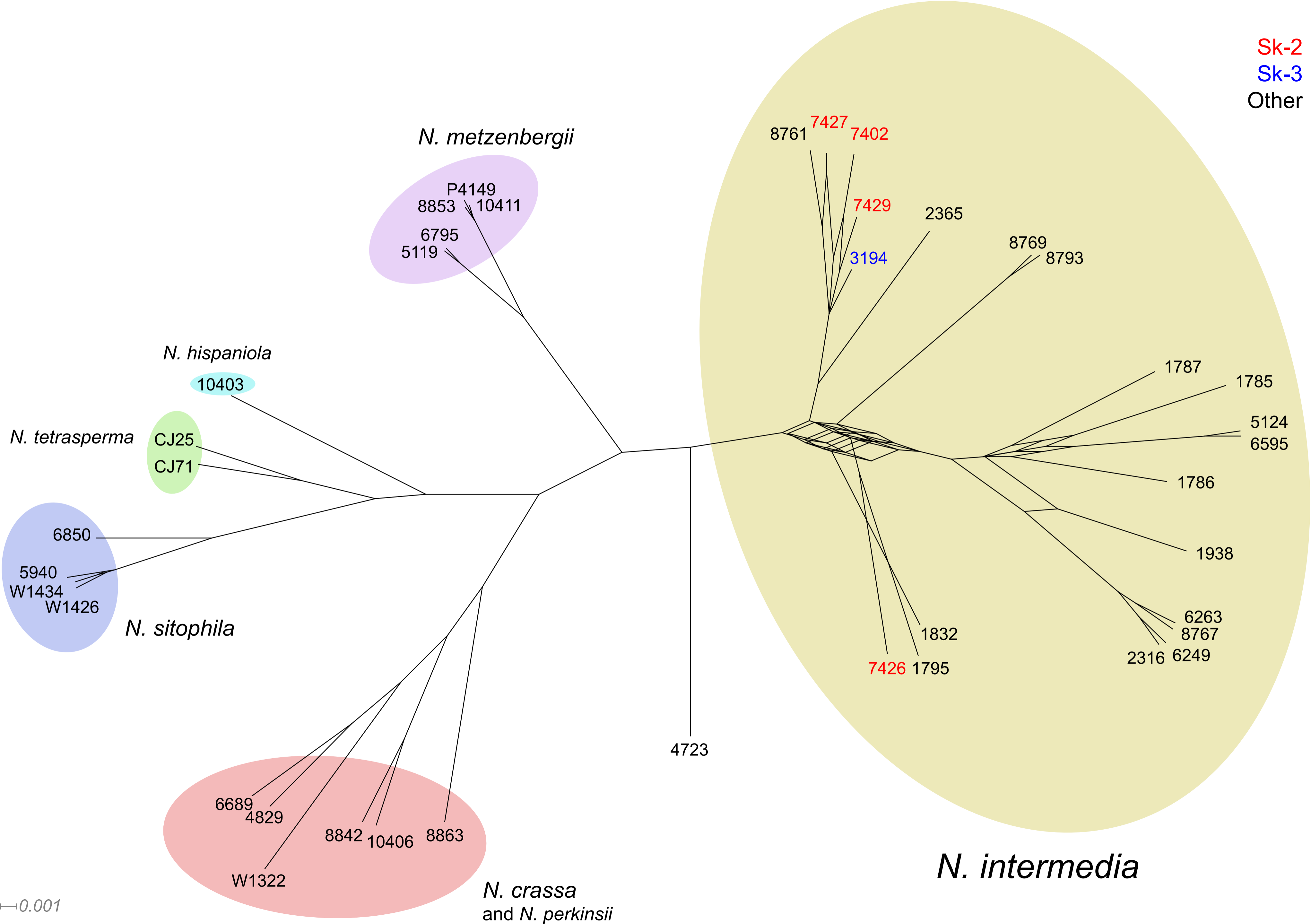

Supplement: Supplementary file 2 — Supplementary Figure 2. Network of the terminal Neurospora clade based on maximum likelihood trees inferred from whole chromosome SNP data from six of the seven chromosomes; Chromosome 3 carries the Sk locus and so was excluded from the analysis. Colored ovals denote species of Neurospora. Note that strain 4723 falls outside all currently delimited species and may represent an undescribed species. Strains carrying Sk‐2 are marked with red text and, the one Sk‐3 strain is marked in blue. Network pruning level was set to 20%. [file EVO-76-2687-s005.pdf]
